# Taurine and oxidative stress in retinal health and disease

**DOI:** 10.1111/cns.13610

**Published:** 2021-02-23

**Authors:** Vanessa Castelli, Antonella Paladini, Michele d’Angelo, Marcello Allegretti, Flavio Mantelli, Laura Brandolini, Pasquale Cocchiaro, Annamaria Cimini, Giustino Varrassi

**Affiliations:** ^1^ Department of Life, Health and Environmental Sciences University of L'Aquila L'Aquila Italy; ^2^ Dompé Farmaceutici SpA L’Aquila Italy; ^3^ Sbarro Institute for Cancer Research and Molecular Medicine and Center for Biotechnology Temple University Philadelphia PA USA; ^4^ Paolo Procacci Foundation Roma Italy

**Keywords:** antioxidants, oxidative stress, retina, retinal disorders, taurine

## Abstract

Retinal disorders are leading causes of blindness and are due to an imbalance between reactive oxygen species and antioxidant scavenger (in favor of pro‐oxidant species) or a disruption of redox signaling and control. Indeed, it is well known that oxidative stress is one of the leading causes of retinal degenerative diseases. Different approaches using nutraceuticals resulted in protective effects in these disorders. This review will discuss the impact of oxidative stress in retinal neurodegenerative diseases and the potential strategies for avoiding or counteracting oxidative damage in retinal tissues, with a specific focus on taurine. Increasing data indicate that taurine may be effective in slowing down the progression of degenerative retinal diseases, thus suggesting that taurine can be a promising candidate for the prevention or as adjuvant treatment of these diseases. The mechanism by which taurine supplementation acts is mainly related to the reduction of oxidative stress. In particular, it has been demonstrated to improve retinal reduced glutathione, malondialdehyde, superoxide dismutase, and catalase activities. Antiapoptotic effects are also involved; however, the protective mechanisms exerted by taurine against retinal damage remain to be further investigated.

## Graphical abstract

Increasing data indicate that taurine supplement may be effective in slowing down the progression of retinal diseases (including glaucoma, AMD, and DR), thus suggesting that taurine can be a promising candidate for the prevention or as adjuvant treatment of these diseases. The mechanism by which taurine supplementation acts is mainly related to the reduction of oxidative stress. Antiapoptotic effects are also involved; however, the protective mechanisms exerted by taurine against retinal damage remain to be further investigated.

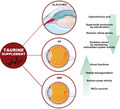

## 1 INTRODUCTION

Oxidative stress (OS) plays a major role in the neurodegenerative process.^1^, ^2^ Retinal cell survival involves redox signaling and a balance between reactive oxygen species (ROS) and antioxidant scavengers to counteract OS injury.^3^, ^4^, ^5^ The retina is susceptible to OS due to its elevated oxygen consumption and exposure to visible light, which can potentiate cellular damage caused by ROS.^6^ Elevated OS levels determine dramatic changes that lead to visual impairment. Age‐related macular degeneration (AMD), diabetic retinopathy (DR), and glaucoma are ocular disorders that can lead to visual loss, and for which the involvement of ROS has been evoked.^7^ Moreover, OS is thought to induce a deficiency of cone photoreceptors in rare inherited retinopathies.^8^ High ROS levels can cause lipid peroxidation which is found at elevated amounts in the photoreceptor cell membrane.^9^ It is noteworthy that products of the oxidation of docosahexaenoic acid (DHA)–containing lipids (CEP‐EPs) are observed at elevated levels both in the eyes and in serum of AMD patients compared with age‐matched controls^9^, ^10^ and are reported as activators of Toll‐like receptor 2 (TLR2) in AMD and in other retinal diseases where ROS exert a role in pathology.^11^ The retina is endowed with an efficient innate immune system that activates three essential pathways: migration of microglia, stimulation of the complement system, and inflammasome assembly in the retinal pigment epithelium (RPE).^12^ For this response, retinal cells are endowed with a variety of immune receptors and mediators such as microbial sensors (TLRs), NOD‐like receptors‐NLRs, RIG‐1 like helicases, cytokines, chemokines, and complement components; all these players are in charge to help the cells to eliminate the insult.^13^ Under OS, the activation of this immune pathway aims to repair tissue homeostasis. Still, under continual stress, the inflammatory system's chronic hyperactivation can determine dramatic tissue changes and damage, resulting in irreversible retinal pathologies, including AMD or DR.^14^, ^15^

The role of ROS as a crucial cause of pathogenic inflammation in chronic disorders has been validated.^2^, ^16^ In fact, it has been reported that pro‐inflammatory cytokines, including TNF‐α, interleukin‐1β, or interferon‐γ, determine ROS increase in RPE cells. Indeed, these pro‐inflammatory cytokines appear to increase in patients’ eyes affected by glaucoma, AMD, DR, or retinal vein occlusion.^3^, ^17^

Taurine (2‐aminoethanesulfonic acid) is a non‐essential amino acid, mostly consumed with the diet.^18^ It is highly present in the eyes, but its physiological role is still uncertain.^19^ Although its role in the retina is unclear, numerous events have been attributed to taurine, involving osmoregulation, antioxidant defense, stress responses, and protein stabilization.^20^ Taurine deficiency leads to photoreceptor degeneration but also to RGC loss. Cone photoreceptors and RGCs appear as the most sensitive cells to taurine deficiency.^21^

It has been reported that taurine levels in animals decrease with aging, and specific electroretinogram changes in rats can be associated with these decreased tissue levels, suggesting that the retina has a reduced capability to counteract the OS.^22^ Exogenous taurine administration may be helpful in counteracting and preventing age‐related alterations in the retina.^22^

Numerous potential targets have been proposed for the neuroprotective effects of taurine, including the restoration of the expression of anti‐ and pro‐apoptotic proteins, its high antioxidant activities, the reduction of calcium influx through voltage‐gated calcium channels, and the reduction of glutamate‐induced excitotoxicity.^19^, ^23^, ^24^, ^25^

Based on the shreds of evidence exposed, this review discusses the role of OS in retinal disorders and the approaches for preventing or counteracting oxidative injuries in retinal tissues, with a particular focus on a promising candidate for their prevention, the taurine.

## 2 THE ORGANIZATION OF THE RETINA

As an extension of the central nervous system, the retina displays similarities to the brain and spinal cord in terms of functionality, anatomy, and immunology.^26^ For instance, the eye shows unique structures and different surface molecules and cytokines and has a specialized immune system similar to those of the brain and spinal cord.^26^ Moreover, in terms of anatomy both the retina and the brain present a barrier that impede circulating pathogens or toxins that could induce infections, but at the same time regulate the passage of vital nutrients.^26^ Blood flow alterations in the brain, due to ischemia for example, are strictly related to blood flow alterations occurring in the eye following by visual impairments.^27^, ^28^

**FIGURE 1:**
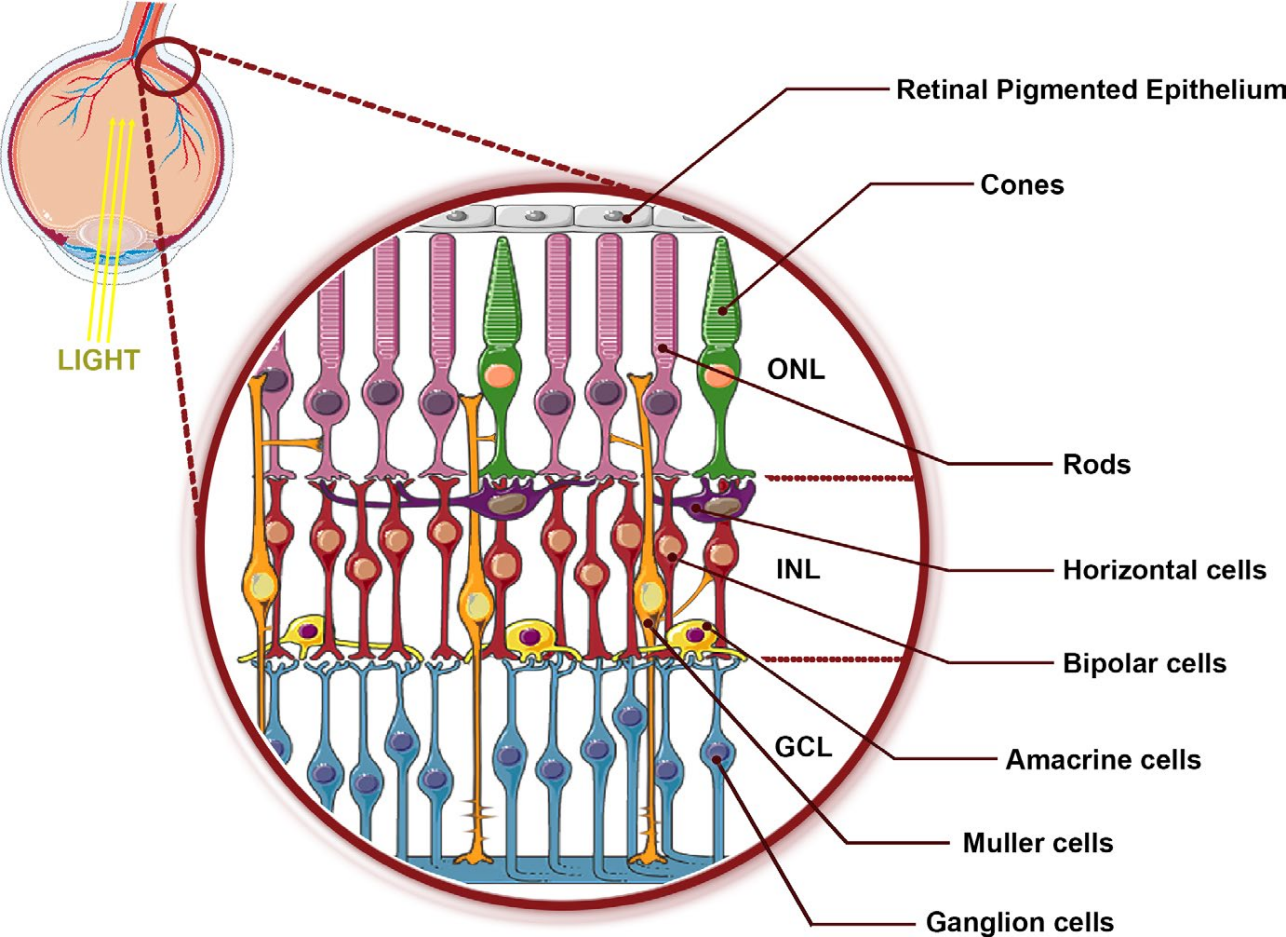
Organization of the mature retina. ONL: outer nuclear layer; INL: inner nuclear layer; and GCL: ganglion cell layer. In the ONL resides the rod and cone photoreceptor cells. The INL contains the amacrine cells, the horizontal cells, and the bipolar cells. The Müller cell body is in this layer, while the processes of the develop into the contiguous layers, expanding all through the thickness of the retina. The GCL mostly contains of the ganglion cells which send their axons out of the eye via the optic disk

Visual processing starts in the retina: a thin, multilayered tissue composed of light‐sensitive neurons lining the back of the vertebrate eye.^29^ The retina elaborates the light produced from visual images through transduction (transferring energy from one form to another) and transfers these data to the brain for the perceptual appreciation of the images.^30^ All vertebrate retinas are composed of three layers of nerve cell bodies, the outer nuclear layer (ONL), the inner nuclear layer (INL), and the ganglion cell layer; and two layers of synapses.^31^, ^32^ ONL is composed of nuclei of photoreceptor cells, which are of two types: rods and cones; the rods and cones synapse with the bipolar cells are in the second layer. INL contains the nuclei and cell bodies of the bipolar, horizontal, and amacrine cells, as well as Müller glial cells.^32^ Bipolar cells spread their extremities to transmit with both the first and third layers. Müller cells are the principal glial cell of the retina and stretch radially across the thickness of the retina. They are responsible for the homeostatic and metabolic support of retinal neurons^31^ (Figure 1).

Rods work mostly in dim light and create no‐colored images. Cones operate in well‐light circumstances and permit the perception of colors and for high‐awareness vision utilized for tasks like reading. The third type of light‐sensitive cells, the ganglion cells, is essential for the entrainment of circadian rhythms and reflexive reactions.^31^, ^33^

Neural signals from the rods and cones are processed by other neurons, whose output brings action potentials in RGCs whose axons generate the optic nerve.^34^ The axons of RGCs in the retina's third layer convey the visual data as coded by the retina to the following synapse point in the visual pathway through the optic nerve.^35^ Amacrine and horizontal cells situated in the INL participate in visual information processing through lateral contacts.^36^ These lateral contacts regulate data transmission through the retina synaptic layers, between the first and second layer and the second and third layer. This intricate system of neurons collects the transduced visual data and processes them by compression, encoding, convergence, and integration.^30^

## 3 OXIDATIVE STRESS IN RETINAL DEGENERATIVE DISEASES

Aging, gene alterations, and excessive exposure to exogenous oxidative stressors (eg, a light exposure) increase oxidative stress in the eye. The relationship between oxidative stress and retinal disorders has been established.^6^, ^7^, ^22^ Oxidative stress shows a crucial role in the onset and progression of retinal disorders, comprising DR, AMD, and glaucoma.^6^, ^8^

### 3.1 Diabetic retinopathy

DR is one of the leading causes of visual impairment and is one of the most common microvascular complications of diabetes.^37^ The polyol pathway is one of the high glucose‐induced metabolic alterations in DR. It converts the glucose in sorbitol, and the reaction is catalyzed by aldose reductase. Sorbitol is then oxidized to produce fructose by sorbitol dehydrogenase. Enhanced polyol pathway in diabetes increases the OS because aldose reductase needs NADPH.^38^ This event may reduce the availability of NADPH for stimulating the intracellular antioxidant, GSH.^39^, ^40^ The production of advanced glycation end products (AGEs) is another pathway causing the detrimental consequences of glucose. AGEs are generated from potent glycating dicarbonyl elements, that is, methylglyoxal and glyoxal.^41^ Chronic hyperglycemia supports enzymatic and non‐enzymatic glycation, inducing altered functions and degradation of intracellular and extracellular proteins by chemical rearrangement and cross‐linking. AGEs are created on amino groups of proteins, lipids, and DNA, leading to intramolecular and intermolecular cross‐links.^41^ In diabetic conditions, the accumulation of AGEs and its receptor, RAGE, are increased in retinal capillary cells.^42^ In the later phases of DR, AGEs are irreversibly generated and deposited in the retinal microvasculature.^42^, ^43^ AGEs enhance nitrative stress in the capillary cells and trigger apoptosis events leading to retinal capillary cell death and pathological consequences.^44^, ^45^

The activation of protein kinase C (PKC) pathway is also involved in the pathogenesis of DR.^46^, ^47^, ^48^ High glucose levels enhance ROS levels, and the synthesis of diacylglycerol, inducing PKC activation.^49^ Activated PKC can determine numerous alterations typical of DR, including enhancing vessel permeability, blood flow, endothelial proliferation, apoptosis, altered hormone and growth factor receptor recycling, increased neovascularization, and regulating various factors as vascular endothelial growth factor, insulin‐like growth factor‐1, and transforming growth factor β.^44^, ^50^ Inhibition of PKC by PKCβ specific inhibitor (LY53331) was able to avoid diabetes‐induced OS.^44^, ^51^ Ohshiro and colleagues showed that lack of PKCβ isoform in mice protected them from diabetes‐induced OS.^52^ These studies linked OS and PKC supported PKC's role in ROS‐mediated diabetic problems.

In diabetes, glucose oxidation is enhanced, generating an elevated voltage gradient across the mitochondrial membrane.^17^ One of the ROS‐induced impairments in mitochondria is the reduced antioxidant defense that may increase retinal cells’ sensitivity to OS. The isoform of SOD in the mitochondria, MnSOD, and GSH are inhibited in diabetic patients and high glucose‐cultured retinal mitochondria.^53^ Mitochondrial impairment also involves injury to mitochondrial DNA,^2^ which also occurs in the diabetic retina.^54^ Enhanced swelling of mitochondrial lipid membranes is detected in the retina of diabetic mice.^54^ The inner mitochondrial membrane includes numerous soluble proteins, including cytochrome c. The release of cytochrome c from mitochondria to the cytoplasm and Bax translocation from the cytosol to mitochondria are enhanced in capillary cells and in the retina in diabetic conditions, events that lead to apoptosis.^55^ Therefore, it is clear that OS can regulate mitochondria activity, causing higher apoptosis in retinal microvasculature; further investigations to define the role of OS‐induced mitochondrial impairments in DR are necessary. Antioxidants may act at various levels; they may prevent ROS formation or scavenge free radicals or enhance antioxidant defenses.

### 3.2 Age‐related macular degeneration

AMD is a multifactorial disorder,^56^ and its pathogenesis remains unclear. Evidence indicates that an intricate interaction of environmental, genetic, and metabolic factors contributes to the pathology of AMD.^57^ AMD is the main reason for visual impairment in the elderly. AMD initially alters the RPE and gradually leads to secondary loss of photoreceptor cells.^58^, ^59^ It is characterized by the degeneration of the macula, described by high number of cone photoreceptors responsible for visual perception and color vision. In AMD, three stages have been recognized: the early stage, the intermediate stage, and the late stage (based on the most recent Three Continent AMD Consortium Severity Scale^60^ and Clinical Classification^61^). The early stage (mild and moderate or severe) is marked by the development of several small drusen or a few medium‐sized drusen^62^; the intermediate stage is characterized by some pigmentary abnormalities and large drusen; and the late stage with several large drusen is characterized by two forms: non‐exudative (“dry form”) and an exudative/neovascular (“wet form”).^63^ The “dry form” is characterized by atrophic alterations in the macula and, clinically, has better conservation of visual acuity than “wet form”.^62^ “Wet” AMD is characterized by atypical blood vessels in the choriocapillaris, which is the formation of new through Bruch's membrane. These vessels lead to bleeding and leakage into the macula and ultimately induce irreversible damage to photoreceptors if untreated.^62^ The “wet form” leads to significant incidence of substantial visual impairment.^64^ End‐stage macular degeneration is the last and irreversible stage, and the patients show visual loss and cannot be longer treated with surgery or ocular injections.

Different pathways are involved in AMD, including OS, apoptosis, the formation of drusen and RPE aberration, immune system activation, senescent failure of homeostatic control, and Bruch's membrane defects.^64^ During aging, antioxidant decreases, and ROS level increases, supporting OS.^2^, ^65^ Furthermore, glutathione S‐transferase‐1 expression level,^66^ macular carotenoids level,^67^ and vitamin E level^68^ are reduced. On the other hand, lipid peroxidation is enhanced,^69^ and lipofuscin,^70^, ^71^ altered mitochondrial DNA in the retina,^72^ and advanced lipid peroxidation and glycation end products^73^ are enhanced. To date, there is no therapy accessible for the “dry type” AMD. In the Age‐Related Eye Disease Study (AREDS), nutraceuticals (AREDS and AREDS2), comprising vitamins C and E, β‐carotene, and zinc, counteracted the disease progression from intermediate to advanced AMD by about 25%.^74^ However, AREDS and AREDS2 supplements do not prevent AMD onset. AREDS investigators followed participants for an additional five years (ten year in total).^75^, ^76^, ^77^

For the “wet‐type,” the anti‐vascular endothelial growth factor (VEGF) antibody is generally used as standard therapy able to ameliorate patients’ visual function.^78^, ^79^ The route of administration is intravitreal injection^80^; but this procedure is invasive and is related to the possibility of infection.^81^, ^82^ Also, the anti‐VEGF antibody is costly; consequently, it is crucial to develop new therapeutic approaches for this pathology.

On light of the pathways involved in AMD, it has been suggested that antioxidant supplement may counteract cellular damage in the retina by reacting with free radicals that are produced in the process of light absorption, thus reducing the risk and progression of AMD.^83^

### 3.3 Glaucoma

Another disorder that leads to irreversible blindness is glaucoma. Open‐angle glaucoma (OAG) is the most common form. OAG is characterized by degeneration of the trabecular meshwork (TM) which rises the intraocular pressure, which in turn, lead to altered axons of RGCs forming the optical nerve, and then progressive concentric damage of the RGCs.^84^, ^85^, ^86^ Degeneration of these cells results in a typical form of the optic disk (cupping) and visual impairment. Furthermore, it is characterized by retinal nerve fiber layer variations and typical visual field defects.^87^ The biological mechanism of glaucoma is poorly recognized, and the factors supporting its progress have not been entirely described.^84^, ^88^ One of the leading risk factors for glaucoma advancement, and the only adaptable factor, is elevated intraocular pressure.^89^ Even if the mechanism is still uncertain, early neuroinflammation is indicated as an underlying trigger of glaucoma pathology.^90^, ^91^ Like AMD, glaucoma is also associated with OS.^92^, ^93^ Indeed, it has been reported that the progressive loss of TM cells in glaucoma patients may be attributed to the long‐term effects of oxidative injury induced by free radicals.^94^, ^95^ This hypothesis was then supported by in vitro and in vivo studies. Human TM in vitro upon hydrogen peroxide showed loss of cellular integrity and reduced cell adhesion.^96^ In vivo, calf TM treated with hydrogen peroxide showed altered the mechanism of drainage of the aqueous humor from the anterior chamber of the eye.^97^ Combined treatment with trophic and antioxidant factors was able to prevent the RGCs death in rats with elevated IOP.^98^ Also, it has been reported that oxidative damage to DNA is considerably higher in the TM of glaucomatous patients compared to controls.^99^ Additionally, in patients, IOP increase and visual impairment are proportional to the amount of oxidative DNA damage affecting TM cells.^100^ In addition, the plasma level of glutathione, an important antioxidant, resulted reduced in glaucoma patients.^101^, ^102^ On light of the exposed evidence, antioxidant therapies could help counteract or reduce this pathology.

## 4 TAURINE IN THE RETINA AND RETINAL DISORDERS

In the retina of mammals, taurine is the most copious amino acid during development and adulthood.^25^ Moreover, the retina appears to be the taurine richest organ,^19^ with concentrations higher than any other ocular structures or the brain,^103^ reaching up to 50 mmol/g tissue in rats. Retinal taurine is provided by Müller cells and RPE, which generally collect taurine and transfer it to photoreceptor cells.^104^ Photoreceptor cells are significantly rich in taurine, and all retinal cells use taurine from the extracellular environment. High‐ and low‐affinity Na^+^ and Cl^−^‐dependent taurine transporters have been reported in the retina. Also, it has been reported that taurine treatment can avoid or counteract retinal neurodegeneration.^23^ Thus, photoreceptors require a sufficient amount of extracellular taurine, depending on their transporter for osmoregulation.^20^, ^105^ However, its function in the retina is still uncertain.

In humans, chronic parenteral nutrition absent of taurine resulted in reduced plasma taurine concentrations and atypical electroretinograms in children and conceivably in adults.^25^, ^106^

A research group investigated if light exposure exacerbated retinal neuronal loss induced by taurine depletion.^107^ As a model, they used albino rats receiving β‐alanine (which causes taurine depletion) in the drinking water, and after one month of treatment, 50% of the rats were subjected to white light (3000 lux).^107^ The results indicated that light exposure under taurine depletion increased photoreceptor degeneration, suggesting that taurine is essential for retinal survival and for light‐induced photoreceptor degeneration.^107^ Consequently, the taurine supplement may counteract degeneration of the retina, particularly S‐cone degeneration or can be useful for the treatment of pathologies for which light may represent an etiologic feature.

**FIGURE 2:**
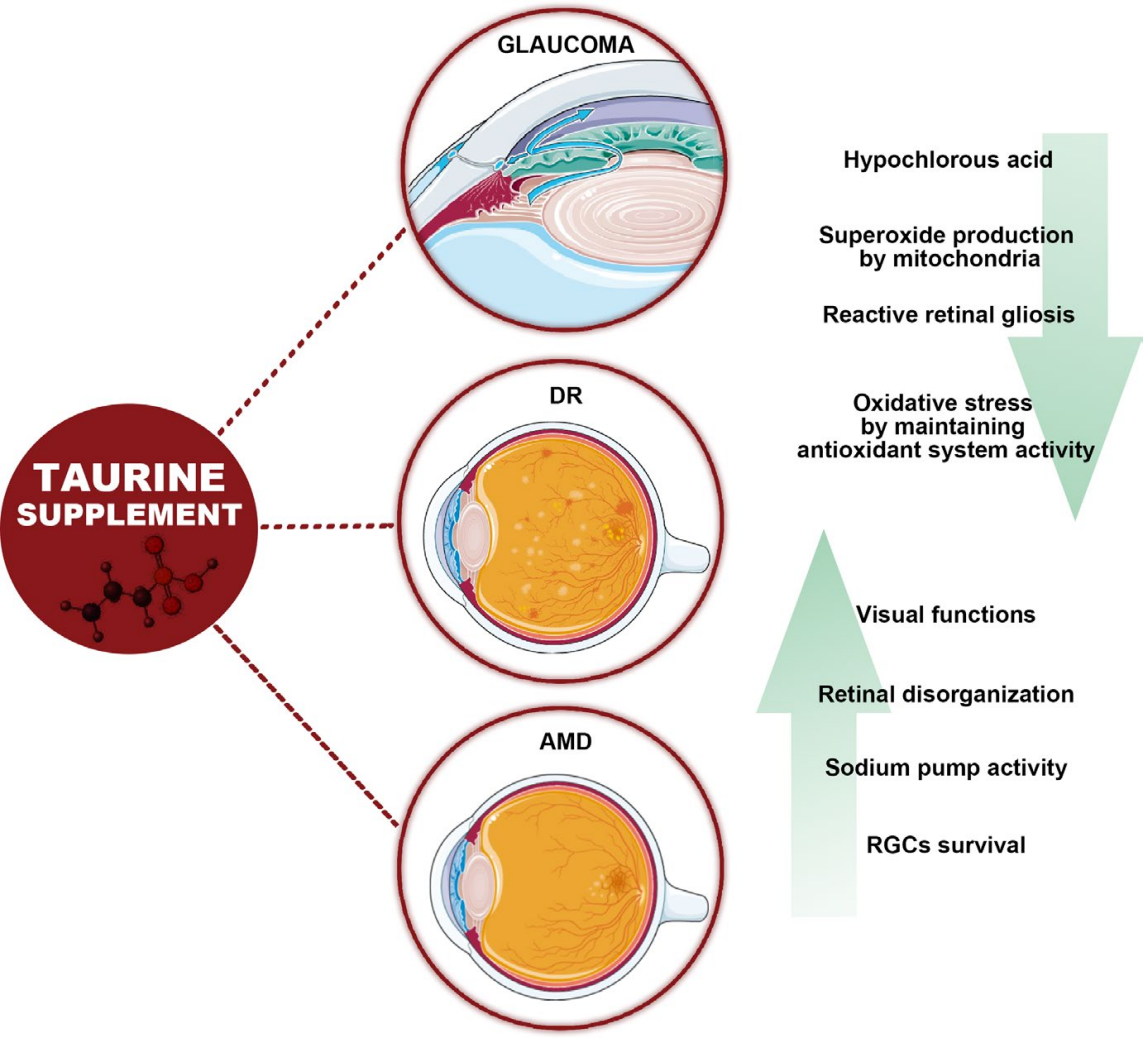
Schematic representation regarding the potential effects of taurine supplement on retinal degenerative disorders. Due to its potential in modulating the oxidative stress, reducing reactive retinal gliosis, and increasing RGC survival as well as sodium pump activity, taurine supplement may represent a therapeutic approach for pathologies like glaucoma, DR, and AMD

One of the main cytoprotective effects of taurine includes its antioxidant activity, mediated by three different processes. First, taurine counteracts the neutrophil oxidant, hypochlorous acid. The product of the reaction between taurine and hypochlorous acid, taurine chloramine, also hinders with the inflammatory pathway.^108^, ^109^ Second, taurine reduces the production of superoxide by mitochondria.^25^, ^110^ Third, mitochondrial ROS can impair antioxidant enzyme activity in balancing OS.^3^, ^110^ Because some antioxidant enzymes are receptive to oxidative injury, taurine may counteract OS by avoiding this enzyme impairment. As mentioned above, taurine deficiency leads to photoreceptor degeneration and also to retinal ganglion cell loss^21^, ^111^; thus, taurine therapy may exert an essential role in preventing retinal degeneration^106^ (Figure 2).

RGC degeneration appears in several retinal disorders leading to visual loss, either as a primary process like in glaucoma or secondary to photoreceptor loss.^86^ However, to date, there is no available treatment directly targeting RGCs neuroprotection. As we mentioned above, taurine seems to be essential for photoreceptors’ survival since its deprivation is related to retinal death. Froger et al^111^ studied the taurine effect on RGCs in vitro models and various animal models of RGC degeneration. Taurine protective effects were evaluated in vitro on primary RCG cultures in serum‐free conditions and on N‐methyl‐D‐aspartate (NMDA)‐treated retinal explants from adult rats. In vivo, two glaucomatous models (mice and rats with vein occlusion) and a model of RP with secondary RGC degeneration (P23H rats) were used. Taurine was administered in the drinking water for 6 days.^111^ Notably, taurine significantly improved RGC survival (+68%) in vitro and partly prevented NMDA‐induced RGC excitotoxicity. In vivo, taurine administration was also able to increase RGC densities in both animal models compared to control groups. This study indicated that enriched taurine nutrition could directly maintain RGC survival, reducing the OS, positively affecting retinal degenerative disorders.^111^

Furthermore, it has been reported that taurine supplementation may be protective and promising therapeutic approach for retinopathies with a chronic cycle, for example, retinitis pigmentosa, an inherited disorder characterized by a progressive degeneration of rod photoreceptors.^112^ Indeed, in a mouse model of N‐methyl‐N‐nitrosourea (MNU)‐induced retinal degeneration, intravenous taurine therapy broadly improved the retinal taurine concentration. Morphological experiments revealed that taurine ameliorated the retinal disorganization in the MNU‐induced animals. Furthermore, taurine was able to ameliorate the vision loss in the MNU‐induced animals, as demonstrated by functional analyses (ie, electroretinogram and optokinetic test). Immunostaining analyses showed that taurine ameliorated both M‐cone and S‐cone populations in the degenerative retinas. Regarding the mechanism, the OS and photoreceptor apoptosis in the degenerative retina were strongly reduced by taurine.^113^

In another interesting study, Arfuzir et al^114^ evaluated taurine neuroprotective properties against glaucoma. In particular, they used endothelin‐1 (ET‐1)‐induced retinal and optic nerve damage. ET‐1 was administered intravitreally to Sprague‐Dawley rats, and taurine was injected as pre‐, co‐, or post‐treatment.^114^ This study suggested that the treatment with taurine, particularly in a preventive regimen, prevented apoptosis of retinal cells induced by ET‐1 and prevented the changes in the morphology of the retina and optic nerve. The protective effect of taurine was also associated with reduced retinal OS. In particular, taurine was able to improve retinal reduced glutathione, malondialdehyde, superoxide dismutase, and catalase activities.^114^

Another group examined the consequences and the main taurine mechanisms on hyperglycemia‐induced variations of Müller cells’ glutamate degradation and uptake. A decreased capability of Müller cells to eliminate glutamate from the extracellular space is critical in the disruption of glutamate homeostasis that appears in the diabetic retina. Taurine substantially reduced the high glucose‐induced reductions in glutamate uptake and counteracted OS induced by high glucose, increasing the antioxidant enzyme events. These results indicate that taurine might control Müller cells’ glutamate uptake and degradation under diabetic conditions through its antioxidant activity.^115^

Recently, Fan et al investigated the role and mechanisms of taurine supplementation (intraperitoneally or intragastrically) in early diabetic retinas using an eight‐week‐old streptozotocin (STZ)‐induced diabetic rats. Taurine protected retinal cone cells as well as RGCs from diabetic attacks by activating retinal taurine transporter, reducing reactive retinal gliosis, enhancing retinal synaptic connections, and reducing retinal cell apoptosis.^116^

Another study examined the chronic taurine treatment vs. a mixture of vitamin E and selenium on biochemical retinal alterations caused by diabetes at different disease stages. STZ‐diabetic rats were treated for 4 months, and taurine was able to significantly reduce retinal OS and to enhance sodium pump activity in experimental diabetes in a dose‐ and time‐dependent manner.^117^ Overall, these findings strengthen the hypothesis that taurine could represent a novel approach for DR.

A recent study evaluated taurine's effects in a family with taurine deficiency (homozygous amino acid substitution in the third transmembrane domain of the taurine transporter SLC6A6). In particular, the authors evaluated taurine levels in the blood and analyzed the fundus and macular with optical coherence tomography after 2 years of taurine supplementation. Interestingly, the retinal degeneration was counteracted, and the vision was clinically ameliorated mostly in the youngest patients (6 years old).^118^

## 5 DISCUSSION AND CONCLUSIONS

It is well known that OS is one of the leading causes of neurodegeneration and retinal degenerative disorders. Indeed, different approaches using antioxidants resulted in protective effects in these disorders.^119^, ^120^, ^121^ Researchers focused on the effects of taurine in retinal disorders. In this review, we reported evidence of the protective role of taurine against retinal functional and morphological injuries in animal and in in vitro retinal disease models, thus implying that taurine may have a therapeutic potential in the treatment of retinal and degenerative disorders. Indeed, increasing data indicate that taurine may be effective in slowing down the progression of retinal diseases, thus suggesting that taurine can be a promising candidate for the prevention or as adjuvant treatment of these diseases. The mechanism by which taurine supplementation acts is mainly reducing the OS, even also antiapoptotic effects are involved; however, the protective effects of taurine against retinal damage still unclear. Further investigations should focus on the way in which taurine protects cells against oxidative stress and toxicity at cellular level and determine whether other treatments can trigger these neuroprotective pathways. Along with other antioxidant molecules, taurine should therefore be strongly reconsidered as a potential treatment for retinal diseases.

## 6 METHODS

Extensive bibliographic research was conducted using the PubMed National Library of Medicine (NIH), Web of Science platform, Google Scholar, and Clinical Key databases. Examples of the search terms used were ‘‘Taurine” ‘‘oxidative stress’’, ‘‘retina’’, ‘‘therapies’’, ‘‘in vitro’’, ‘‘in vivo’’ “health retina” “retinal degeneration”. For screening, a restriction was made to those articles published in the last 10 years and preferably in English. Priority was given to prospective studies and reviews with a clear and well‐described methods section. In addition, a secondary search of the bibliography of the articles finally selected was carried out to detect possible omissions. For the analysis of all relevant publications, consensus meetings were held with all the authors.

## CONFLICTS OF INTEREST

The authors declare they have no conflict of interest with the material reported in this article.

## AUTHOR CONTRIBUTIONS

VC wrote the manuscript with support from AC and GV. AP, MA, FM, LB, and PC substantively revised it. MdA prepared all the figures. All authors contributed to the final manuscript. All authors have read and agreed to the published version of the manuscript.

## Data Availability

Not applicable.
